# Acteoside Improves Muscle Atrophy and Motor Function by Inducing New Myokine Secretion in Chronic Spinal Cord Injury

**DOI:** 10.1089/neu.2018.6000

**Published:** 2019-05-28

**Authors:** Atsushi Kodani, Takahiro Kikuchi, Chihiro Tohda

**Affiliations:** Division of Neuromedical Science, Institute of Natural Medicine, University of Toyama, Toyama, Japan.

**Keywords:** axonal growth, chronic spinal cord injury, muscle atrophy, myokine, pyruvate kinase M2

## Abstract

Chronic spinal cord injury (SCI) is difficult to cure, even by several approaches effective at the acute or subacute phase. We focused on skeletal muscle atrophy as a detrimental factor in chronic SCI and explored drugs that protect against muscle atrophy and activate secretion of axonal growth factors from skeletal muscle. We found that acteoside induced the secretion of axonal growth factors from skeletal muscle cells and proliferation of these cells. Intramuscular injection of acteoside in mice with chronic SCI recovered skeletal muscle weight reduction and motor function impairment. We also identified pyruvate kinase isoform M2 (PKM2) as a secreted factor from skeletal muscle cells, stimulated by acteoside. Extracellular PKM2 enhanced proliferation of skeletal muscle cells and axonal growth in cultured neurons. Further, we showed that PKM2 might cross the blood–brain barrier. These results indicate that effects of acteoside on chronic SCI might be mediated by PKM2 secretion from skeletal muscles. This study proposes that the candidate drug acteoside and a new myokine, PKM2, could be used for the treatment of chronic SCI.

## Introduction

In spinal cord injury (SCI), the traumatic disruption of the neuronal connections between the central and peripheral nervous system that terminate to skeletal muscle leads to severe motor dysfunction. The time course of SCI is loosely defined by a hyper-acute phase to a chronic phase.^1^ Mechanical insult at the spinal cord ruptures local neurons and glia at the lesion site within minutes to hours (hyper-acute phase). A secondary damage, such as apoptosis of neurons and glia and leakage of the blood–spinal cord barrier (BSCB), follows immediately after. The infiltration of neutrophils, lymphocytes, macrophages, and microglia are increased at this stage until roughly 7 days after injury (acute phase). Glial scar formation and chondroitin sulphate proteoglycan (CSPG) production are enhanced in the following period, until approximately 4 weeks after injury (sub-acute phase). From 1 month post-injury and onwards, namely the chronic phase, cavity formation^2^ and CSPG accumulation^3^ severely progress, whereas M2 macrophage infiltration in the epicenter is reduced.^3^ Spontaneous axonal sprouting hardly occurs in the chronic phase, which renders motor function and sensory function untreatable in this time-window.

In acute and subacute phases of SCI, several options of treatments are experimentally effective; in animal models, overexpression of neurotrophic factors^4,5^ or transplantation of neural stem cells at the lesion site promote functional recovery via axonal regeneration.^6^ However, the recovery of motor function by such treatments is insufficient in cases of chronic SCI.^7,8^ Considering that most SCI patients are already in the chronic phase, the discovery of useful therapeutic strategies for chronic SCI is an urgent issue.

Although the majority of SCI studies have focused on the nervous system and inflammatory cells in the spinal cord, we targeted skeletal muscle atrophy, as a characteristic finding in the chronic phase. A longitudinal study showed that skeletal muscle atrophy progresses in a manner dependent on the time after injury in humans.^9,10^ Disuse of skeletal muscle and loss of motor neurons^11^ synergistically induce muscle atrophy. On the contrary, exercise, such as treadmill training in rodents^12^ and robotic gait training in humans,^13^ was shown to slightly improve motor function when applied during the chronic phase. The authors of these studies proposed task-specific and use-dependent neuronal plasticity and modification as reasons of the training-elicited improvements.^12^ However, we considered that an important hint was ignored in these results of exercise-elicited recovery of motor function. Forced movement of skeletal muscle might stimulate the muscle itself, which in turn might affect the related neuronal circuit. This might occur via the interesting actions of myokines.

Skeletal muscle is an endocrine organ that secretes several factors to maintain its homeostasis, which are collectively termed myokines and include cytokines, peptides, and growth factors.^14,15^ Representative myokines, such as insulin growth factor 1 (IGF-1), brain-derived neurotrophic factor (BDNF), cathepsin B, and irisin, are considered to affect remote organs, including the central nervous system.^16,17^ However, in clinical studies, 12- to 16- week exercise training did not increase IGF-1 serum levels in recruited patients with SCI in the chronic phase (more than 1 year after injury),^18^ although a similar training program significantly increased these levels in healthy subjects.^19^ This suggests that even if skeletal muscle has myokine-releasing properties, atrophied muscle in the chronic phase of SCI hardly responds to exercise. Therefore, myokine-mediated improvement of motor function after SCI has not been demonstrated yet.

We considered that exercise is not the only way to enhance myokine secretion. Thus, we hypothesized that stimulation of skeletal muscle with drugs rather than exercise might activate the release of known or yet unknown myokines, with the notion that the identification of such drugs and new myokines would pave a new way of therapy for SCI.

By our previous screening approach, we identified acteoside as a stimulant of myokine secretion (unpublished data). Acteoside was reported to exert anti-inflammatory,^20,21^ anti-oxidant,^22^ and neuroprotective^23,24^ effects. However, acteoside-induced myokine secretion and SCI recovery have not been investigated. In this study, we aimed to clarify the effects of acteoside on chronic SCI and the mechanism by which it induces myokine secretion.

## Methods

All animal experiments and protocols were carried out in accordance with the Guidelines for the Care and Use of Laboratory Animals of the Sugitani Campus of the University of Toyama. The approval numbers for the animal experiments are A2013INM-1 and A2016INM-3. All efforts were made to minimize the number of animals used.

### Primary cortical neuron culture

Embryos of ddY mice (Japan SLC, Japan) were obtained at the 14th day of gestation. The dura was removed, and cerebral cortices were isolated, minced, and dispersed. Cells were cultured on 8-well chamber slides (Corning, NY), coated with poly-D-Lysine (PDL; 5 μg/mL; Sigma-Aldrich, St. Louis, MO), at a density of 1.5 × 10^4^ cells/well, in Neurobasal medium (Thermo Fisher Scientific, Waltham, MA) containing 12% horse serum, L-glutamine, and 0.6% D-glucose at 37°C in a humidified incubator with 10% CO_2_. Five hours after seeding, the medium was replaced with fresh Neurobasal medium containing 2% B-27 supplement (Thermo Fisher Scientific), 0.6% D-glucose, and 2 mmol/L L-glutamine. For CSPG coating, culture dishes were coated with 5 μg/mL PDL (Sigma-Aldrich) and 2 μg/mL aggrecan (Sigma-Aldrich) in Hank's buffered salt solution (HBSS; Thermo Fisher Scientific) overnight at 37°C.

### Primary skeletal muscle cell culture

Post-natal Day 3 ddY mice (Japan SLC, Hamamatsu, Japan) were decapitated under anesthesia with isoflurane (FUJIFILM Wako Pure Chemical, Osaka, Japan). Hindlimbs were cut and the skin was peeled off. Isolated skeletal muscle from the whole hindlimb was minced, then dissociated in HBSS containing 1 mg/mL collagenase (Sigma-Aldrich), 0.02 mg/mL dispase I (Sigma-Aldrich), and 2.5 mM CaCl_2_ for 1 h. The obtained myocytes were grown in Myocyte Medium (Promo Cell, Heidelberg, Germany) in several culture plates without coating; cells were plated at a cell density of 1.0 × 10^4^ cells/well in white-walled 96-well plates (Greiner Japan, Tokyo, Japan), 2.0 × 10^4^ cells/well in eight-well chamber slides, or 6.0 × 10^5^ cells/dish in 100-mm dishes (Corning). Cells were incubated at 37°C in a humidified incubator with 10% CO_2_.

### Preparation of conditioned media (CM)

Mouse skeletal muscle cells were treated with acteoside (Extrasynthese, Lyon, France) at doses of 1, 100, and 1000 nM or with vehicle solution at culture Day 1. Three days after treatment, cells were washed two times with and kept in fresh serum-free Dulbecco's Modified Eagle's Medium (Thermo Fisher Scientific). CM was collected and filtered using a 0.45-μm pore membrane filter (Advantec, Tokyo, Japan) to remove cell debris. For axonal growth assay, the CM was mixed with Neurobasal medium containing B-27 supplement in a one-to-one ratio. For silver staining and Western blotting, the CM was concentrated using a centrifugal filter device (Amicon Ultra-4, 3K; Merck Millipore, Darmstadt, Germany), and the protein concentration in the CM was quantified using the Pierce 660 nm Protein Assay (Thermo Fisher Scientific).

### Immunocytochemistry for cultured cortical neurons

After treatment with the CM, recombinant pyruvate kinase isoform M2 (PKM2; 0.01, 0.1, 1, and 10 ng/mL), or vehicle solution, neurons were fixed with paraformaldehyde for 90 min. Axons were immunostained with the mouse monoclonal anti-phosphorylated neurofilament-H (pNF-H) antibody (1:250; Covance Inc., Princeton, NJ). Neuronal cell bodies were immunostained with the rabbit polyclonal anti–microtubule-associated protein 2 (MAP2) antibody (1:2000; Abcam, Cambridge, UK). As secondary antibodies, we used Alexa Fluor-488-conjugated goat anti-mouse immunoglobulin G (IgG) and Alexa Fluor-568-conjugated goat anti-rabbit IgG (1:300; Thermo Fisher Scientific). Nuclear staining with 1 μg/mL 4′6-diamino-2-phenylindole (DAPI) was performed at room temperature. Eight to 23 images per well were captured using a fluorescent microscope (Axio Cell Observer Z1; Carl Zeiss, Oberkochen, Germany), at a field of 432 μm × 323 μm or 863 μm × 645 μm. The length of pNF-H–positive axons was measured using MetaMorph software (Molecular devices, San Jose, CA), which automatically traces and measures neurite lengths. The total axonal length in a given image was divided by the number of MAP2- and DAPI-double positive cells to calculate the axonal length per neuron.

### Immunocytochemistry for skeletal muscle cells

Skeletal muscle cells were fixed with paraformaldehyde for 90 min after treatment with acteoside or vehicle solution. Myotubes were immunostained with the rabbit polyclonal anti-desmin antibody (1:250; Covance Inc.), followed by the Alexa Fluor 568-conjugated goat anti-rabbit IgG antibody (1:300). Images, 10–13 per well, were captured using a fluorescent microscope (Axio Cell Observer Z1, Carl Zeiss), at a field of 432 μm × 323 μm or 863 μm × 645 μm. Desmin-positive cells were counted using MetaMorph software.

### Silver staining

Concentrated CM samples (1 or 3 μg/lane) were electrophoresed on SDS-PAGE (10% separating gel), and separated proteins were stained using SilverQuest Silver Staining kit (Thermo Fisher Scientific). The target protein bands were cut out, in-gel digested with trypsin (Promega, Fitchburg, WI), and identified using nano liquid chromatography–mass spectrometry (LC-MS/MS; Japan Bio Services, Saitama, Japan) and MASCOT database search.

### Western blotting

Concentrated CM samples (3 μg/lane) were electrophoresed on SDS-PAGE (10% separating gel) and transferred to a nitrocellulose membrane. After blocking with 5% nonfat dry milk for 1 h at room temperature, the membrane was incubated with the rabbit monoclonal antibody against PKM2 (1:1000; Cell signaling, Danvers, MA) in 0.1% Tween 20-containing TBS (T-TBS) overnight at 4°C. After four times washing with T-TBS, the membrane was incubated with horse-radish peroxidase (HRP)-conjugated goat anti-rabbit IgG antibody (1:2000; Santa Cruz Biotechnology, Dallas, TX) for 2 h at room temperature. After four times washing with T-TBS, the membrane was soaked in a detection solution (ECL prime reagent; GE healthcare, Pittsburgh, PA) for 2 min, and bands were detected using a luminescence image analyzer (LAS 4000, Fuji Film, Tokyo, Japan). CS analyzer (ATTO, Tokyo, Japan) was used to quantify the luminescence intensity of each band.

### SCI experiment

Ten-week-old female ddY mice (SLC) were used for the SCI experiment. Mice were housed in a plastic cage (23 cm × 16 cm × 12 cm) under a 12-h light/dark cycle (light period: 7:00–19:00), at constant temperature and humidity (22 ± 2°C, 55 ± 10%). Water and solid food were allowed *ad libitum.* Mice were anaesthetized by intraperitoneal (i.p.) administration of a mix of three types of anesthetics: 0.675 mg/kg of medetomidine (ZENOAQ, Koriyama, Japan), 3.6 mg/kg of midazolam (Sandoz, Tokyo, Japan), and 4.5 mg/kg of butorphanol (Meiji Seika Pharma, Tokyo, Japan). After laminectomy at the level of T11, contusion injury was performed onto the exposed L1 segment of the spinal cord by drop of a 13.5-g rod (tip diameter of 1 mm) from a 3-cm height (approximately 13.5 kdyn). Mice were allowed to recover on a hot pad to maintain body temperature, and 0.75 mg/kg atipamezole (ZENOAQ) was injected to antagonize medetomidine.

Acteoside is known to reach the skeletal muscle after oral administration,^25^ where it is highly distributed; thus, we considered it should also reach the muscle by an intramuscular injection. By using this administration route, we expected that acteoside would reach the atrophied muscle directly and in high concentration. Thirty days after SCI, acteoside or vehicle solution was injected intramuscularly into the right and left biceps femoris muscles (three times/week). Behavioral observations were performed during the pre-injection (30 days) and injection period (62 days), once every 7 days. Acteoside was injected to the vastus lateralis of the right and left hindlimbs (0.1 mg/limb), three times a week.

### Behavioral evaluation

For behavioral scoring after surgery, mice were placed in an open cage (50.0 cm × 42.5 cm × 15.0 cm) and observed while moving free for 3 min. The motor function of hindlimbs was evaluated using the Basso Mouse Scale (BMS)^26^ and Toyama Mouse Score (TMS).^27^ Movements of the left and right hindlimbs were evaluated independently.

### Wet weight of muscles and immunohistochemistry for 5-hydroxytryptamine (5-HT)–positive axonal tracts

After the behavioral observations, mice were anaesthetized by administration of a mix of three anesthetics (0.75 mg/kg of medetomidine [ZENOAQ], 4.0 mg/kg of midazolam [Sandoz], and 5.0 mg/kg of butorphanol [Meiji Seika Pharma], i.p.) and perfused with ice-cold saline and 4% paraformaldehyde in phosphate-buffered saline to fix the tissues.

The bilateral biceps femoris and tibial anterior were excised. The tendons were carefully cut. The muscle wet mass was weighed using an electronic analytical balance with a precision of 0.1 mg.

The spinal cords were dissected at the T10-L1 and L2-L6 level, soaked in 30% sucrose, embedded with cryomold 3 (Sakura Finetech Japan, Tokyo, Japan), and stored at −30°C until use. Spinal cords were then cut into 14-μm sagittal sections using a cryostat (CM 3050S; Leica Microsystems, Wetzlar, Germany). The sections were post-fixed in 4% paraformaldehyde and immunostained at 4°C for 24 h with the rabbit polyclonal anti-serotonin (5-HT; 1:500; ImmunoStar, Hudson, WI) and mouse monoclonal anti-glial fibrillary acidic protein (GFAP; 1:1000; Sigma-Aldrich) antibodies. Alexa Fluor 488-conjugated goat anti-rabbit IgG antibody (1:400; Life Technologies) and Alexa Fluor 594-conjugated goat anti-mouse IgG antibody (1:400; Life Technologies) were used as secondary antibodies. Nuclear staining with 1 μg/mL DAPI was performed at room temperature. Images were obtained using a fluorescence microscope (BZ-ZX700/BZ-X710, Keyence, Osaka, Japan) and quantified using Image J (National Institutes of Health). The area surrounded by the GFAP-positive area was defined as the injury area, where the glial scar was formed. The three most center slides of serial sections were selected for quantification. Areas of 5-HT–positive axons in the gray matter, located at rostral and caudal positions 2 mm away from the injury site were quantified.

### Immunohistochemistry for synaptophysin on Fluoro-Gold–positive motor neurons

Seven days before the end of behavioral observations, 5% Fluoro-Gold solution (Sigma-Aldrich) was injected into the tibial anterior and gastrocnemius muscles of right and left hindlimbs (2.5 μL/site). Spinal cord sections at the L2-L6 level were prepared as described above for the 5-HT immunohistochemistry. Sections were post-fixed in 4% paraformaldehyde, immunostained with the mouse monoclonal anti-synaptophysin (1:500; Sigma-Aldrich) and rabbit monoclonal anti-Fluoro-Gold (1:500; Fluorochrome, Denver, CO) antibodies at 4°C for 24 h, followed by Alexa Fluor 488-conjugated goat anti-mouse IgG (1:400; Life Technologies) and Alexa Fluor-Cy5-conjugated anti-rabbit IgG (1:50; Life Technologies) secondary antibodies, and counterstained with DAPI. Images were obtained using a fluorescence microscope (BZ-ZX700/BZ-X710; Keyence) and quantified using Image J (National Institutes of Health). The number of Fluoro-Gold–positive motor neurons was counted. Synaptophysin-positive puncta on Fluoro-Gold–positive motor neuron cell bodies were quantified. All slices containing Fluoro-Gold–positive neurons were quantified.

### PKM2 levels in skeletal muscle, plasma, and brain

PKM2 levels in the whole brain, biceps femoris, and plasma were quantified using ELISA (enzyme-linked immunosorbent assay) Kit for mouse PKM2 (Cloud-Clone, Katy, TX) according to the manufacturer's protocol. In brief, acteoside was injected to the vastus lateralis of the right and left hindlimbs (0.1 mg/limb) of healthy ddY mice (female, 10-weeks-old). After 1, 3, and 6 h, mice were perfused with ice-cold saline, and biceps femoris and plasma were isolated. After the frozen biceps femoris tissue was ground using a multi-bead shocker (Yasui Kikai, Osaka, Japan), lysates were prepared in radioimmunoprecipitation assay buffer (RIPA) buffer, and protein concentration was adjusted to 5 μg/100 μL for ELISA. Plasma was collected using Amber BD Microtaine Plasma Separator Tube (Becton, Dickinson and Company, Franklin Lakes, NJ), and its protein concentration was also adjusted to 5 μg/100 μL for ELISA.

Recombinant mouse PKM2 (10 μg) or saline was intravenously injected in healthy ddY mice (female, 10-weeks-old). After 20 min, mice were perfused with ice-cold saline and the brain was isolated. After chopping the tissues with microscissors, brain lysates were prepared in RIPA buffer, and protein concentration was adjusted to 5 μg/100 μL for ELISA.

### Statistical analysis

Statistical comparisons were performed using one-way analysis of variance (ANOVA) with *post hoc* Bonferroni tests, repeated measures two-way ANOVA with *post hoc* Bonferroni tests, or unpaired two-tailed *t*-test, using GraphPad Prism 6 (GraphPad Software, San Diego, CA). Split plot and *post hoc* Tukey's honestly significant difference (HSD) test was performed using JMP Pro 13 (SAS Institute Japan, Tokyo, Japan). A *p* value of <0.05 was considered significant. The data are presented as the mean ± standard error of the mean (SEM).

## Results

### Acteoside treatment enhances cultured skeletal muscle cell proliferation

**FIG. 1.**
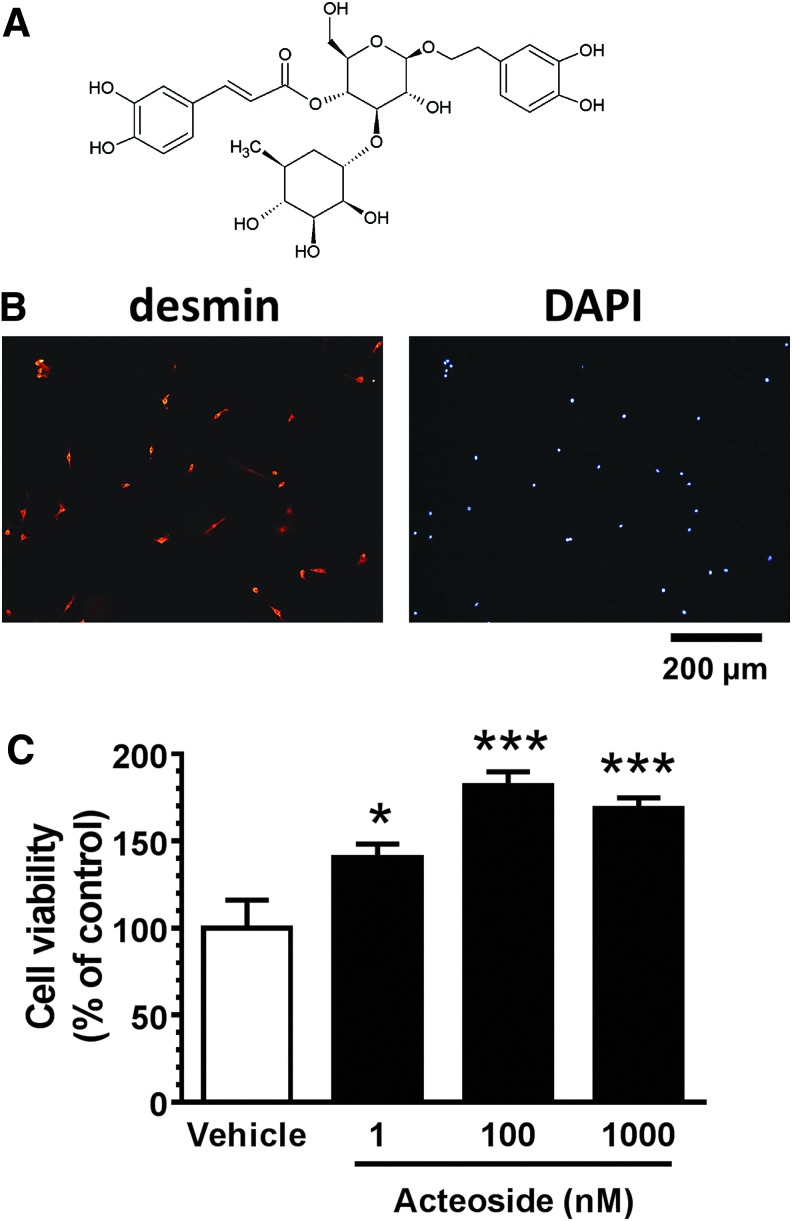
Acteoside effects on skeletal muscle cell proliferation. **(A)** Structure of acteoside. **(B)** Cultured skeletal muscle cells were immunostained for desmin and counterstained with 4′6-diamino-2-phenylindole (DAPI). Scale bar: 200 μm. **(C)** Primary cultures of mouse skeletal muscle cells were treated with acteoside (1, 100, 1000 nM) or vehicle solution for 3 days. After treatment, cell proliferation was evaluated by Cell Titer Glo reagent. *n* = 6; *p < 0.05; ***p < 0.001 vs. vehicle; one-way analysis of variance *post hoc* Bonferroni test. Data are shown as mean ± standard error. Color image is available online.

Skeletal muscle cells were treated by acteoside (Fig. 1A) or vehicle solution after 1 day in culture. Three days after drug treatment, we evaluated cell proliferation. Myocytes were identified as desmin-positive cells. Almost all cells were desmin-positive (Fig. 1B). The proliferation of skeletal muscle cells significantly and dose-dependently increased by incubation with acteoside (1, 100, or 1000 nM) but not with vehicle (Fig. 1C).

### Acteoside-treated skeletal muscle cell CM promotes axonal growth

**FIG. 2.**
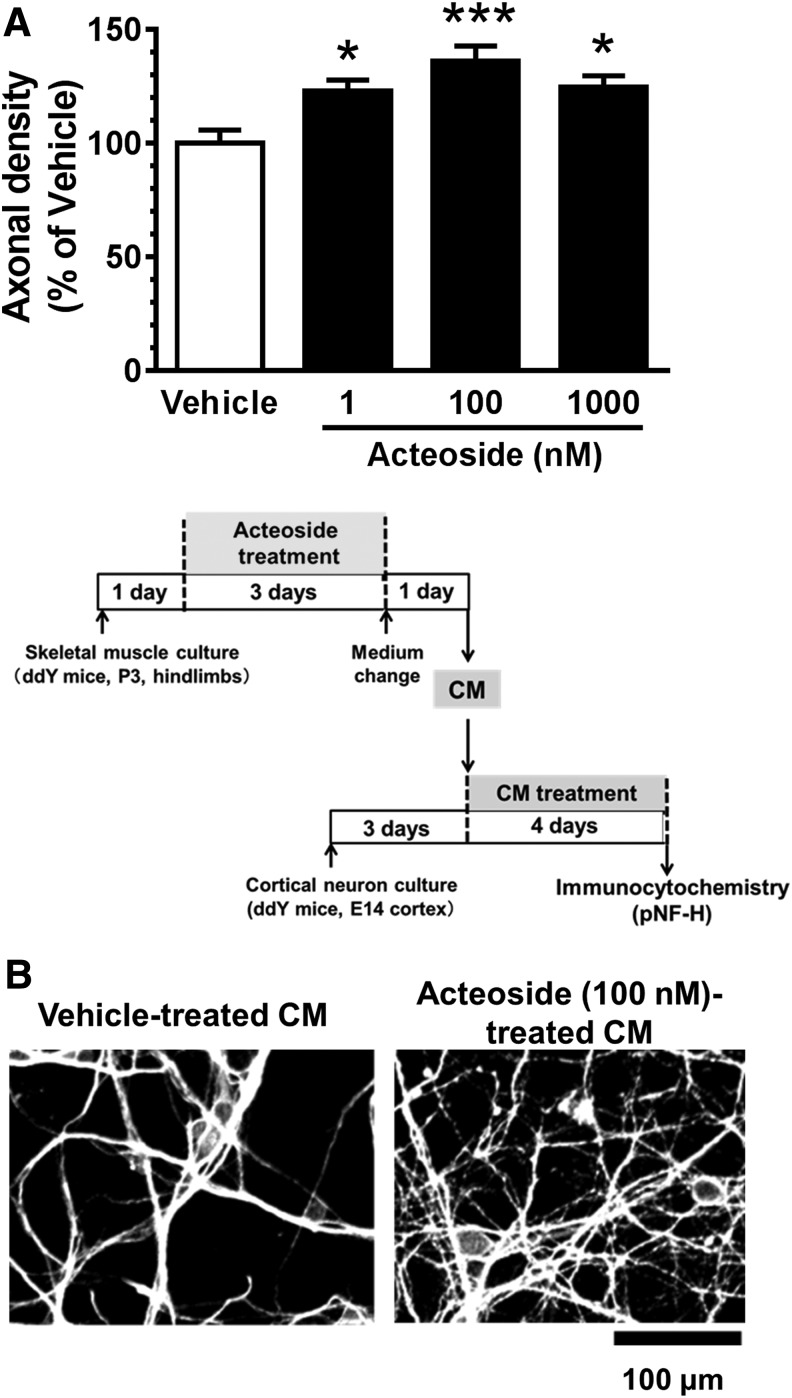
Activity of conditioned medium (CM) from acteoside-treated skeletal muscle cells on axonal growth. **(A)** Primary cultured mouse skeletal muscle cells were treated with acteoside (1, 100, 1000 nM) or vehicle solution at culture Day 1. After 3 days, the medium was changed to a serum- and acteoside-free medium. Twenty-four hours after, CM was collected. Separately prepared mouse cortical neurons were treated with this CM. After 4 days of CM treatment, axonal densities were evaluated by immunocytochemistry using phosphorylated neurofilament-H (pNF-H) and MAP2 antibodies. The densities of pNF-H–positive axons per neuron were quantified. *n* = 14–16 captured images. **(B)** Representative images showing axons of cortical neurons treated by CM. Scale bar: 100 μm. *p < 0.05; ***p < 0.001 vs. vehicle; one-way analysis of variance, *post hoc* Bonferroni test. Data are shown as mean ± standard error.

To investigate whether acteoside promotes the secretion of axonal growth factors from skeletal muscle cells, mouse cortical neurons were treated with CM from cultured skeletal muscle cells that had been treated with acteoside (1, 100, or 1000 nM) at culture Day 3. Four days after the CM treatment, axonal densities were evaluated by immunocytochemistry using pNF-H and MAP2 antibodies. The axonal densities were significantly higher in cortical neurons cultured with the CM from skeletal muscle cells treated with any dose of acteoside than with that from cells treated with vehicle (Fig. 2A, 2B). This result suggests that acteoside enhances the secretion of some axonal growth factors from skeletal muscle cells.

### Intramuscular injection of acteoside improves locomotion in mice with contusive SCI at the chronic phase

**FIG. 3.**
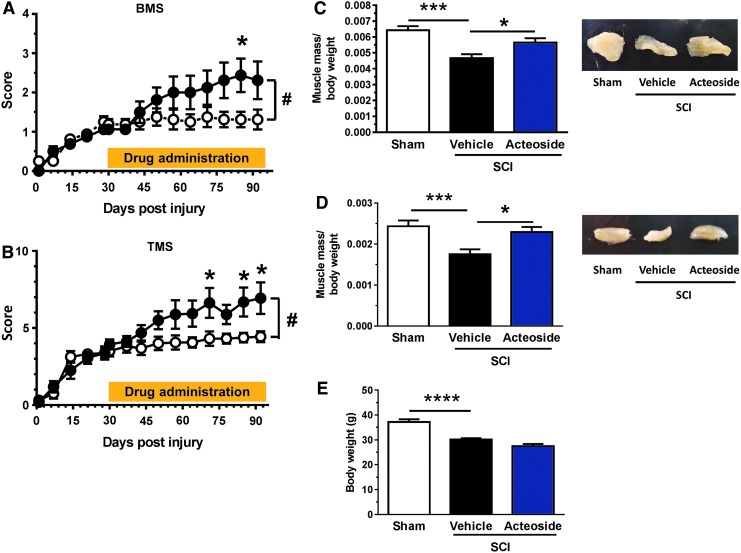
Acteoside effects on locomotor function of mice with contusive spinal cord injury (SCI). Thirty days after SCI, acteoside or vehicle solution was injected intramuscularly into the right and left biceps femoris muscles of mice (3 times/week). Behavioral observations were performed during the pre-injection (30 days) and injection period (62 days), once every 7 days. Hindlimb motor function of mice with SCI was evaluated using the Basso Mouse Scale (BMS; **A**) and Toyama Mouse Score (TMS; **B**). Acteoside group: *n* = 8 mice; *n* = 16 hindlimbs. Vehicle group: *n* = 8 mice, *n* = 16 hindlimbs. *p < 0.05 vs. vehicle; repeated measures two-way analysis of variance (ANOVA), *post hoc* Bonferroni test in (A) and (B). #*p* < 0.0001 vs. vehicle; drug × day interaction by repeated measures two-way ANOVA in (A) and (B). Data are shown as mean ± standard error (SE). Biceps femoris **(C)** and tibial anterior **(D)** muscles were dissected to measure wet weights at the end of the behavioral observations. Muscle mass was normalized by the body weight at that time. Representative muscle tissues are shown. *p < 0.05; ***p < 0.001; ****p < 0.001 vs. SCI/vehicle; one-way ANOVA, *post hoc* Bonferroni test. Acteoside group, *n* = 8 mice, *n* = 16 skeletal muscles. Vehicle group: *n* = 8 mice, *n* = 16 skeletal muscles. Sham: *n* = 6 mice, *n* = 12 skeletal muscles. Data are shown as mean ± SE. Color image is available online.

To examine the effect of acteoside on locomotor function of mice with chronic SCI, we intramuscularly injected acteoside or vehicle solution (saline) to the vastus lateralis of paralyzed hindlimbs. Drug injection was initiated on Day 30 post-injury and continued for 62 days. The hindlimb motor function was evaluated by using BMS^26^ and TMS.^27^ Behavioral observations were performed once every 7 days. Repeated measures two-way ANOVA revealed a significant time × drug interaction when comparing the acteoside- and vehicle-treated SCI mice [BMS: F(14, 420) = 3.91, *p* < 0.0001, Fig. 3A; TMS: F(14, 420) = 4.19, *p* < 0.0001, Fig. 3B]. The scores were significantly higher in acteoside- than in vehicle-treated SCI mice at 55 days after drug administration, for the BMS, and at 31, 55, and 62 days for the TMS.

### Skeletal muscle atrophy in chronic SCI mice is ameliorated by acteoside injection

To evaluate the effect of acteoside on skeletal muscle atrophy in mice with chronic SCI, the wet weights of the biceps femoris (Fig. 3C) and tibial anterior (Fig. 3D) muscles were measured at the end of the drug treatment. The weights of both muscles were significantly lower in vehicle-treated SCI mice than in sham-operated mice. In contrast, the weights were significantly higher in the acteoside-treated than the vehicle-treated SCI group. Body weights were not different between these two groups, although both groups exhibited reduced body weights compared with the sham-operated group (Fig. 3E). Those results indicate that skeletal muscle atrophy in mice with chronic SCI is ameliorated by intramuscular injection of acteoside.

### Acteoside increases axonal density at the caudal lesion site in mice with chronic SCI

**FIG. 4.**
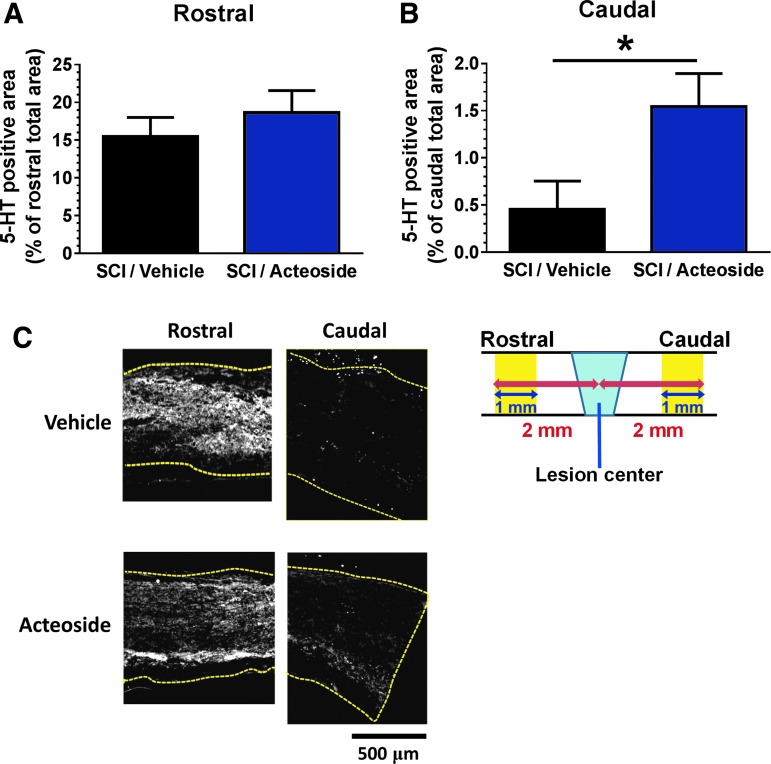
Acteoside effects on axonal growth at the lesion site of mice with chronic spinal cord injury (SCI). Acteoside or vehicle solution was intramuscularly injected into the right and left femoral bicep muscles for 62 days, three times a week, starting at Day 30 after SCI. Fluorescent immunostaining was performed in spinal cord sections using the 5-hydroxytryptamine (5-HT) antibody. 5-HT–positive regions in the gray matter were quantified at rostral **(A)** and caudal **(B)** sites, 2 mm away from the lesion center. **(C)** Representative images of 5-HT immunostaining at the rostral and caudal sites in vehicle solution- and acteoside-treated mice. Yellow dotted lines indicate outlines of the spinal cords. In a scheme, yellow areas are quantified regions in rostral and caudal sites. Scale bar: 500 μm. *p < 0.05, split plot, *post hoc* Tukey's honest significant difference test. SCI/Vehicle group: *n* = 4 mice. SCI/Acteoside group: *n* = 3 mice. Data are shown as mean ± standard error. Color image is available online.

Raphespinal tract is serotonergic and one of the major descending tracts that modulate excitability of motor neurons.^28^ We evaluated axonal growth around the lesion site in acteoside-treated mice. Although the density of 5-HT–positive fibers was not changed in the rostral side of the lesion's epicenter (Fig. 4A), in the caudal side it was significantly higher in acteoside-treated than in vehicle-treated SCI mice [F(1, 19) = 5.6465, *p* = 0.0282, split plot *post hoc* Tukey's HSD test; Fig. 4B].

### Acteoside increases presynaptic density on motor neurons at the caudal lesion site in mice with chronic SCI

**FIG. 5.**
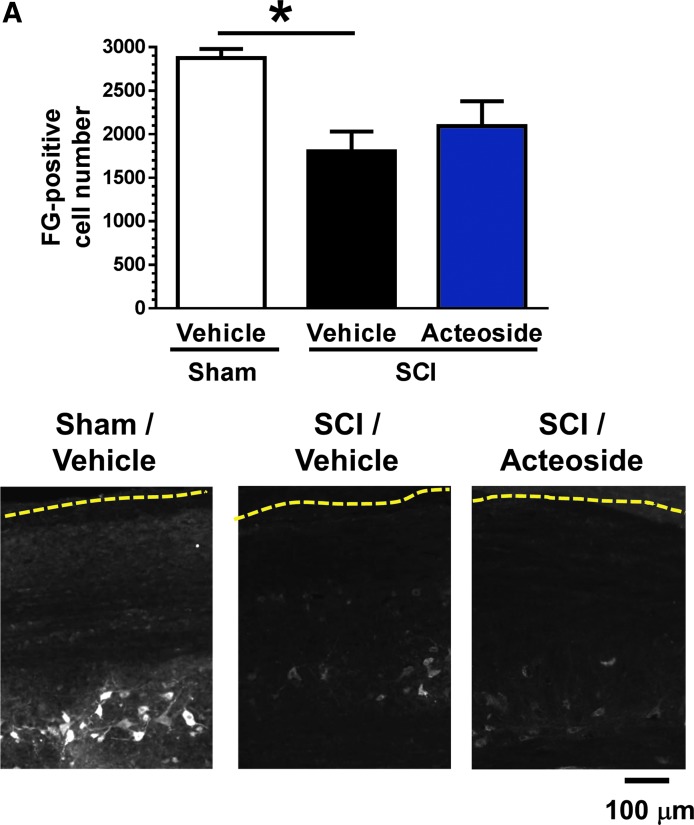

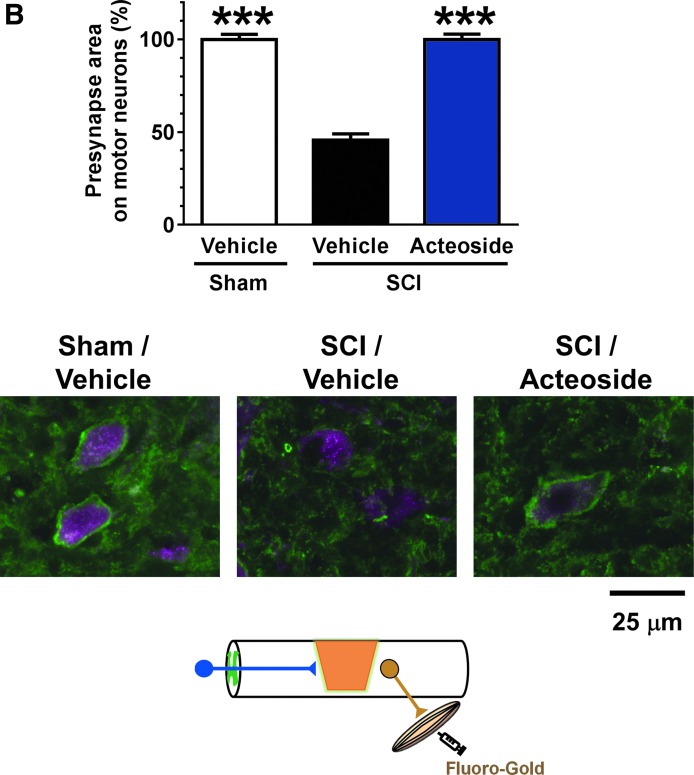
Numbers of motor neurons and presynaptic sites on motor neurons that terminate to hindlimb muscles. (**A)** Top: Quantification of Fluoro-Gold–labeled motor neurons in the caudal site of the lesion center (L2-L6 level). Sham group: *n* = 3 mice. SCI/Vehicle group: *n* = 5 mice. SCI/Acteoside group: *n* = 5 mice. Data are shown as mean ± standard error. Bottom: Representative photos of sections from each group are shown. White color indicates Fluoro-Gold–positive cells. Scale bar = 100 μm. **(B)** Top: Synaptophysin-positive puncta on motor neurons that were labeled by Fluoro-Gold were quantified. *p < 0.05, ***p < 0.001 vs. SCI/vehicle; split plot, *post hoc* Tukey's honest significant difference test. Sham group: *n* = 1322 neurons (3 mice). SCI/Vehicle group: *n* = 825 neurons (3 mice). SCI/Acteoside group: *n* = 1254 neurons (4 mice). Data are shown as mean ± standard error. Bottom: Representative photos of sections from each group are shown. Green and magenta indicate synaptophysin-positive and Fluoro-Gold–positive fluorescence, respectively. Scale bar = 25 μm. Color image is available online.

A retrograde tracer, Fluoro-Gold, was injected in the tibial anterior and gastrocnemius muscles of right and left hindlimbs. Those muscles are regulated by motor neurons distributed in L2-L4 cord segments.^29^ Thus, we counted Fluoro-Gold–positive motor neurons in those segments. The number of Fluoro-Gold–labeled motor neurons was significantly decreased in vehicle-treated SCI mice, while acteoside treatment did not significantly change this number (Fig. 5A). Pre-synaptic areas on motor neurons that terminate to the tibial anterior and gastrocnemius muscles were detected and quantified as synaptophysin-positive puncta on Fluoro-Gold–positive motor neuron cell bodies. Synaptophysin-positive puncta were fewer in vehicle-treated SCI mice than in sham-operated mice. Acteoside treatment completely reversed this effect of SCI [(F(2, 156.7) = 96.0840, *p* < 0.0001, split plot *post hoc* Tukey's HSD test; Fig. 5B].

### PKM2 is secreted from cultured skeletal muscle cells

**FIG. 6.**
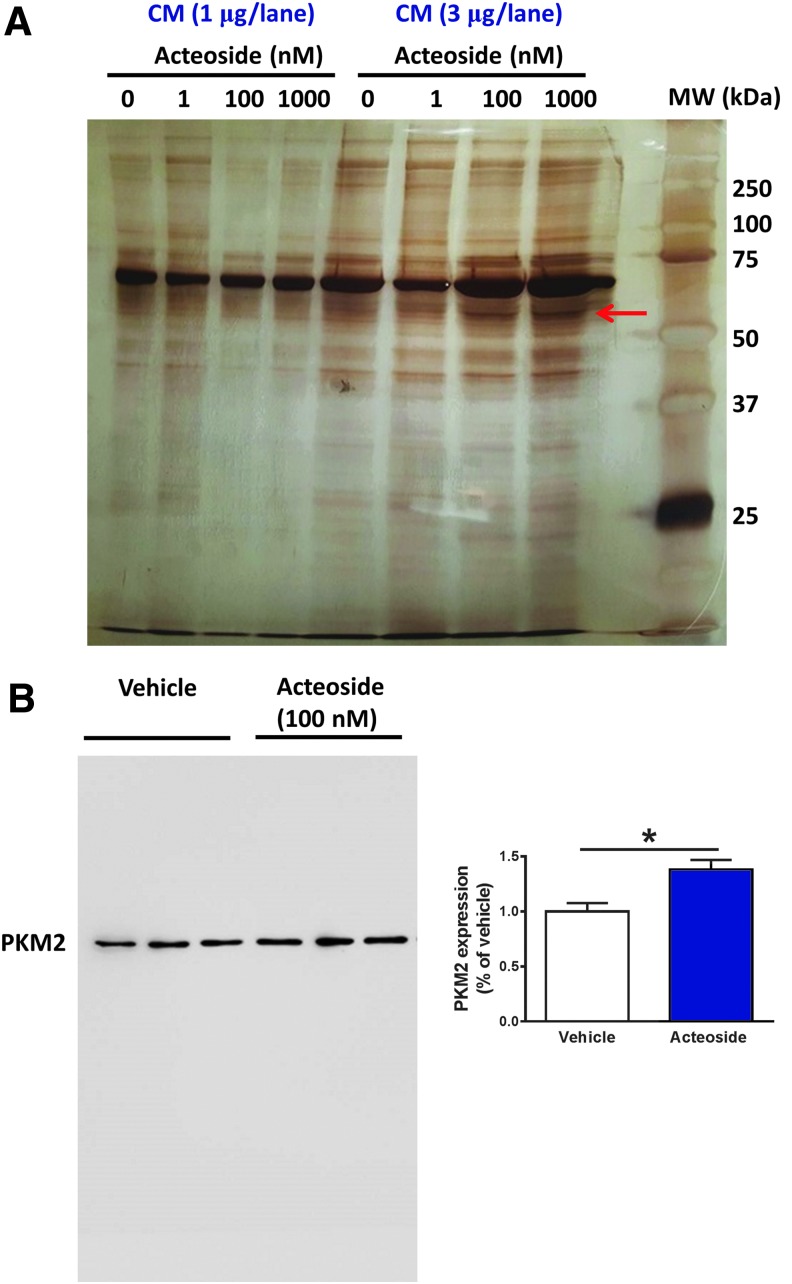
Identification of myokines secreted from skeletal muscle cells by acteoside. **(A)** Primary cultured skeletal muscle cells were stimulated by acteoside (1, 100, 1000 nM) or vehicle solution for 3 days. The medium was then changed to a serum- and acteoside-free medium. Twenty-four hours later, the conditioned medium (CM) was collected. CM samples (1 μg and 3 μg) were separated on SDS-PAGE and silver stained. The red arrow points to a protein that was increased in acteoside-treated CM (3 μg). This protein was identified as the pyruvate kinase isoform M2 (PKM2) by nano liquid chromatography/mass spectrometry analysis. **(B)** Western blotting for PKM2 confirmed that the increased protein in CM was PKM2. *n* = 3. *p < 0.05 vs. vehicle; unpaired two-tailed *t*-test. Data are shown as mean ± standard error. Color image is available online.

Based on the results shown in Figure 2, we hypothesized that some axonal growth factors are secreted from skeletal muscle cells upon acteoside treatment. To address this issue, we treated cultures of primary mouse skeletal muscle cells with acteoside (1, 100, 1000 nM) or vehicle for 3 days and collected the medium (CM). Separation on SDS-PAGE and silver staining showed that two protein bands of approximately 60 and 75 kDa increased dose-dependently (Fig. 6A). Nano LC-MS/MS analysis showed that these bands corresponded to PKM2 (identified amino acid coverage: 38%) and periostin (identified amino acid coverage: 8%), respectively. To confirm secretion of those proteins by muscle cells, we quantified their levels in the CM by Western blotting. Although PKM2 was higher in the CM of acteoside-treated muscle cells (Fig. 6B), periostin levels were not significantly changed (data not shown). Therefore, we focused on PKM2 as a new myokine candidate.

### Extracellular PKM2 acts on skeletal muscle cells and neurons

**FIG. 7.**
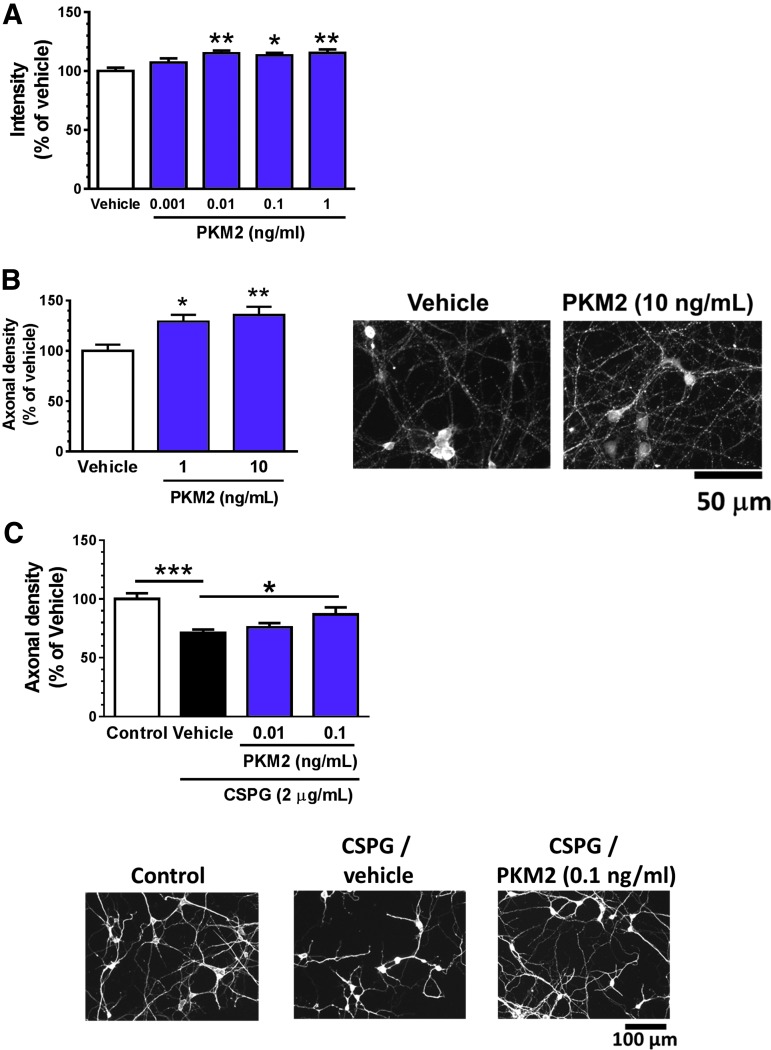
Effects of pyruvate kinase isoform M2 (PKM2) on the proliferation of skeletal muscle cells and axonal growth in cortical neurons. **(A)** Primary cultured mouse skeletal muscle cells were treated by mouse recombinant PKM2 (0.001, 0.01, 0.1, and 1.0 ng/mL) or vehicle solution for 3 days. After the treatment, cell proliferation was evaluated by Cell Titer Glo reagent. **(B)** Mouse cortical neurons were treated with recombinant mouse PKM2 (1 and 10 ng/mL). Four days after the treatment, axonal densities were evaluated by immunocytochemistry using phosphorylated neurofilament-H (pNF-H) and microtubule-associated protein 2 (MAP2) antibodies. The densities of pNF-H–positive axons per neuron were quantified. *n* = 8 captured images. Representative images show axons of cortical neurons treated with recombinant PKM2. Scale bar: 50 μm. **(C)** Mouse cortical neurons were cultured on chondroitin sulphate proteoglycan (CSPG; 2 μg/mL)–coated dishes for 3 days. The cells were treated with recombinant mouse PKM2 (0.01 and 0.1 ng/mL) or vehicle solution. After additional incubation for 4 days, neurons were fixed and immunostained for pNF-H and MAP2. The density of pNF-H–positive axons per neuron was quantified. *n* = 20–23 captured images. Representative images show axons of cortical neurons treated with recombinant PKM2. Scale bar: 100 μm. *p < 0.05; **p < 0.01; ***p < 0.001 vs. vehicle; one-way analysis of variance, *post hoc* Bonferroni test. Data are shown as mean ± standard error. Color image is available online.

PKM2 catalyzes the transfer of a phosphate group from phospho-enolpyruvate to pyruvate.^30^ Besides the pyruvate kinase activity of PKM2 as a cytosolic protein, it also acts as an extracellular protein. PKM2 secretion from cancer cells and induction of cancer cell proliferation by extracellular PKM2 were reported.^31^ However, muscle-mediated PKM2 secretion and its function have not been demonstrated. To reveal the function of extracellular PKM2, we examined its effect on muscle cell proliferation. Mouse skeletal muscle cells were treated by recombinant PKM2 or vehicle for 3 days. Cell proliferation was significantly increased by PKM2 at doses of 0.01, 0.1, and 1 ng/mL (Fig. 7A). Further, we treated mouse cortical neurons with recombinant PKM2 or vehicle solution for 4 days. Compared with vehicle, PKM2 significantly increased the axonal densities of cortical neurons at doses of 1 and 10 ng/mL (Fig. 7B). Next, the activity of PKM2 on axonal growth was investigated using CSPG as a substrate. CSPG is a major axonal growth inhibitor secreted from astrocytes at the lesion site.^32^ Mouse cortical neurons were seeded on CSPG-coated dishes and treated with recombinant PKM2 or vehicle solution for 4 days. Axonal density was significantly lower in vehicle solution–treated neurons cultured on CSPG substrate than in those cultured without. In contrast, PKM2 significantly increased the axonal density at a dose of 0.1 ng/mL, even in neurons cultured on CSPG substrate (Fig. 7C).

**FIG. 8.**
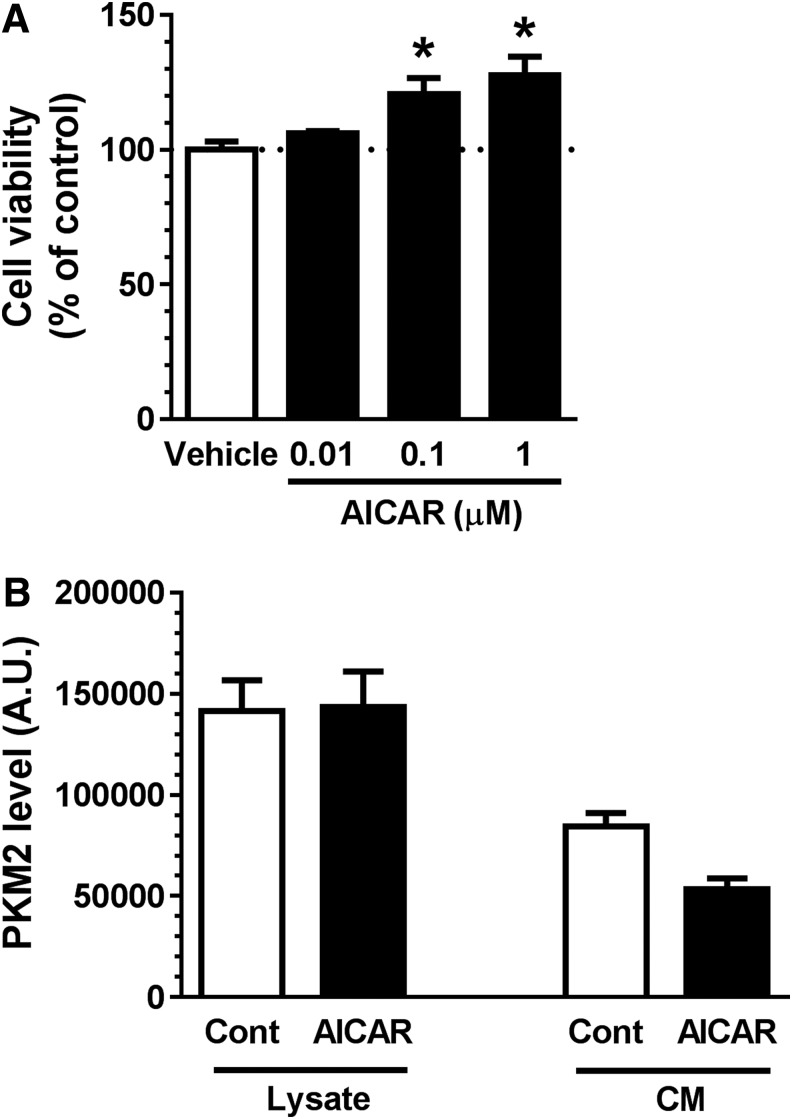
Effects of 5-aminoimidazole-4-carboxamide 1-beta-D-ribofuranoside (AICAR) on skeletal muscle cell proliferation and pyruvate kinase isoform M2 (PKM2) expression and secretion in skeletal muscle cells. **(A)** Cultures of primary skeletal muscle cells were treated with AICAR (0.01, 0.1, and 1 μM) or vehicle solution. After 2 days, cell proliferation was evaluated using Cell Titer Glo reagent. *n* = 4 (AICAR) or 8 (Vehicle). *p < 0.05 vs. vehicle; one-way analysis of variance, *post hoc* Bonferroni test. Data are shown as mean ± standard error. **(B)** Cultures of primary skeletal muscle cells were treated with AICAR (1 μM) or vehicle solution for 3 days. Twenty-four hours later, the conditioned medium (CM) was collected. Muscle cell lysate (8 μg) and CM sample (8 μg) were separated on SDS-PAGE. PKM2 expression levels were quantified by Western blotting. *n* = 3.

Secretion of myokines is generally enhanced by exercise.^14^ To examine whether this was the case for PMK2, we used the exercise-mimicking reagent, 5-aminoimidazole-4-carboxamide 1-beta-D-ribofuranoside (AICAR).^33^ AICAR increased myocyte proliferation (Fig. 8A) but did not enhance secretion of PKM2 from myocytes (Fig. 8B). This result indicates that PKM2 secretion is specifically induced by acteoside and might be independent to exercise.

### PKM2 transfers from blood to the brain

**FIG. 9.**
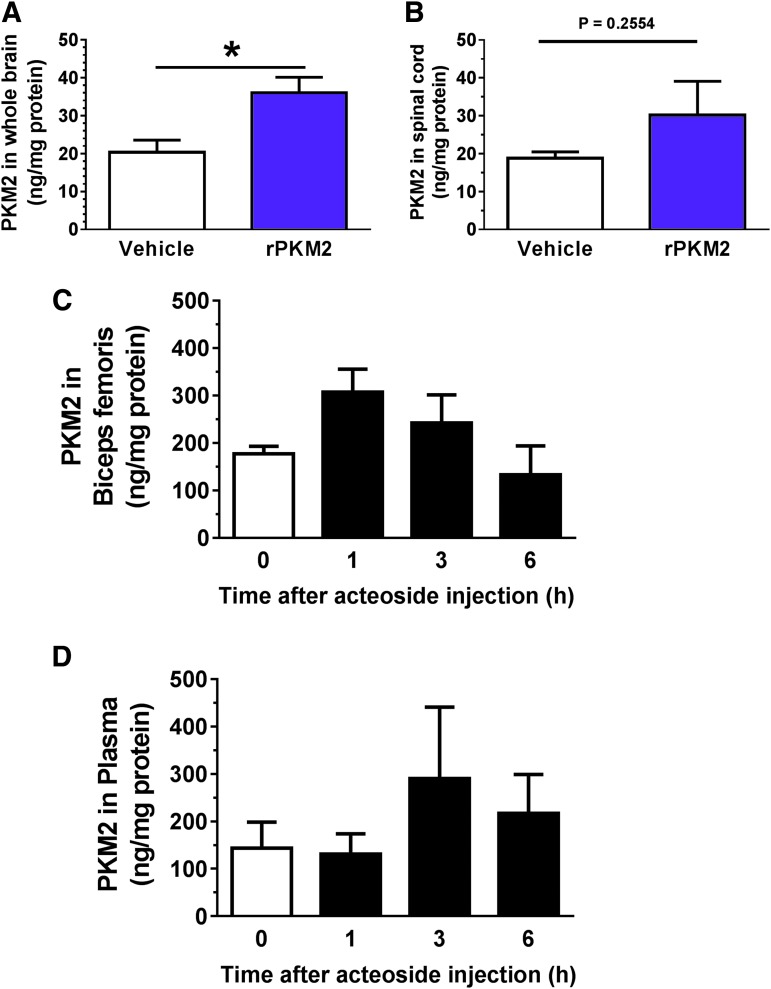
Pyruvate kinase isoform M2 (PKM2) transfers from blood to the central nervous system. **(A, B)** Recombinant mouse PKM2 (10 μg) or vehicle solution was intravenously injected to each mouse. Twenty minutes later, PKM2 concentration in the whole brain (A) and spinal cord (T10-L1) (B) were evaluated. *p < 0.05 vs. vehicle; unpaired two-tailed *t*-test. Recombinant PKM2 group: *n* = 4 mice; vehicle group: *n* = 3 mice. Data are shown as mean ± standard error. PKM2 levels in biceps femoris **(C)** and plasma **(D)** were quantified at 0, 1, 3, and 6 h after intramuscular injection of acteoside (0.2 mg/2 limbs). Acteoside group: *n* = 3 mice; vehicle group: *n* = 3 mice. Data are shown as mean ± standard error. Color image is available online.

To investigate whether PKM2 is transferred from blood to the central nervous system, mouse recombinant PKM2 (10 μg) or vehicle solution was intravenously injected into healthy mice. Twenty minutes after injection, the whole brain and spinal cord (T10-L1) were dissected after complete blood removal. Brain and spinal cord lysates were prepared for ELISA. PKM2 concentration was significantly higher in the brain after injection of recombinant PKM2 than of vehicle solution (Fig. 9A). PKM2 concentration in the spinal cord also was increased in mice injected with recombinant PKM2, but these results did not reach significance (Fig. 9B). Within such a short period (20 min after injection), an increase in *PKM2* transcription is unlikely. Thus, these results suggest that PKM2 transfers from the blood to the central nervous system.

We next quantified PKM2 levels after intramuscular injection of acteoside in biceps femoris and plasma by ELISA. PKM2 in biceps femoris increased at least 1 h after acteoside injection, and then gradually decreased until 6 h (Fig. 9C). In contrast, PKM2 in plasma peaked at 3 h after the injection (Fig. 9D). These results suggest that intramuscular injection of acteoside elevated PKM2 levels first in the muscle and then in plasma. As shown in Figure 9D, the highest PKM2 concentration in plasma was about 300 ng/mg of plasma protein at 3 h after acteoside injection. We calculated that the presumed amount of PKM2 in the blood 3 h after acteoside injection would be 22 ng. Generally, delivery of substances into the brain is restricted by the blood–brain barrier (BBB); thus, the presumed dose of PKM2 was below the detection limit of ELISA. Therefore, a high dose of recombinant PKM2 (10 μg) was injected for the experiments shown in Figure 9A and 9B.

## Discussion

Two major issues in chronic SCI are the untreatable axonal disruption and the severe atrophy of skeletal muscle; our study showed that both may be improved by acteoside, via PKM2 secretion. In this study, we found that acteoside injection into skeletal muscle at the chronic phase of SCI recovers locomotor dysfunction and skeletal muscle atrophy in mice, despite the fact that our contusion model was quite severe; less than grade 2 at 30 days post-injury. A series of similar experiments by our group have shown that acteoside injection, starting at 43 days post-injury, also significantly improves hindlimb motor function (data not shown). Further, acteoside injection enhanced axonal growth in 5-HT–positive cells and synaptogenesis at the caudal side of the lesion center. Although the number of motor neurons was reduced by SCI, consistently to what is shown in human patients,^11^ acteoside did not increase that number significantly. In contrast, synaptogenesis on motor neurons terminating to the tibial anterior and gastrocnemius muscles was increased by acteoside. Taken together, these data suggest that acteoside injection may enhance the extension of at least raphespinal cord tracts and termination of axons, possibly including descending tracts and interneurons.

Importantly, we showed that acteoside stimulates the secretion of PKM2 from skeletal muscle and that PKM2 promotes proliferation of skeletal muscle cells and axonal growth. Our results indicate that PKM2 is a new myokine that activates skeletal muscle and neurons and that its secretion is enhanced by acteoside stimulation. Although drug administration alone is generally insufficient to recover motor function in patients with chronic SCI, acteoside might be a promising therapeutic drug for SCI treatment by inducing “muscle-mediated axonal growth.”

Further, we found that PKM2 might cross the BBB and BSCB despite its large molecular weight. After exercise, transcription of several myokines is increased in the brain.^17,34^ However, there is no clear evidence on whether myokines transfer to the brain. PKM2 may be a new type of myokine that reaches the brain and potentially the spinal cord. The advantages and significance of PKM2 penetration in the central nervous system should be clarified in future. The mechanism by which PMK2 crosses the BBB and BSCB might be similar to that in the case of IGF-1 (MW: 8.5 kDa), which crosses the BBB by transcytosis.^35^ The elongation of raphespinal tracts might be induced by PKM2 stimulation both at the level of cell bodies and axonal terminals. PKM2 also might affect interneurons, although it should be investigated further.

It is interesting that extracellular PKM2 has dual effects on neurons and skeletal muscle. Although intracellular PKM2 is known as a glycometabolic enzyme that converts phosphoenolpyruvate to pyruvic acid, extracellular PKM2 seems to have other properties, independent of its enzymatic role. For example, extracellular PKM2 was reported to stimulate the epidermal growth factor (EGF) receptor and its downstream phosphorylation in breast cancer cell lines, mediated by protein kinase B (Akt) and extracellular signal-regulated kinase (ERK).^31^ Although the contribution of EGF receptor to axonal growth in neurons is controversial,^36^ its stimulation was shown to enhance muscle satellite cell activation.^37^ Therefore, it is necessary to identify the direct target molecules of extracellular PKM2 in neurons and skeletal muscle cells, which might or might not include EGF receptor.

This study did not clarify how PKM2 secretion is elevated by acteoside. To address this issue, the signaling pathway mediating the effects of acteoside in muscle cells should be investigated. Several findings implicate acteoside in diverse signaling pathways. For example, acteoside-mediated neuroprotection against amyloid β-induced cell death in PC12 cells is mediated by ERK and PI3K/Akt pathways.^23^ In cancer cells, acteoside directly binds to and inhibits protein kinase C.^38^ Moreover, binding to and inhibition of caspase-3 of acteoside were found in neurons.^39^ In inflammatory cells stimulated with lipopolysaccharide, acteoside promotes nuclear factor κB inhibition.^21^ Inhibition of calcium influx by acteoside has also been reported.^40,41^ Since the PKM2 secretion mechanism has not been determined in previous studies, it is completely unknown which of the acteoside-induced pathways are involved in the secretion of PKM2. Future experiments would be required to determine this.

**FIG. 10.**
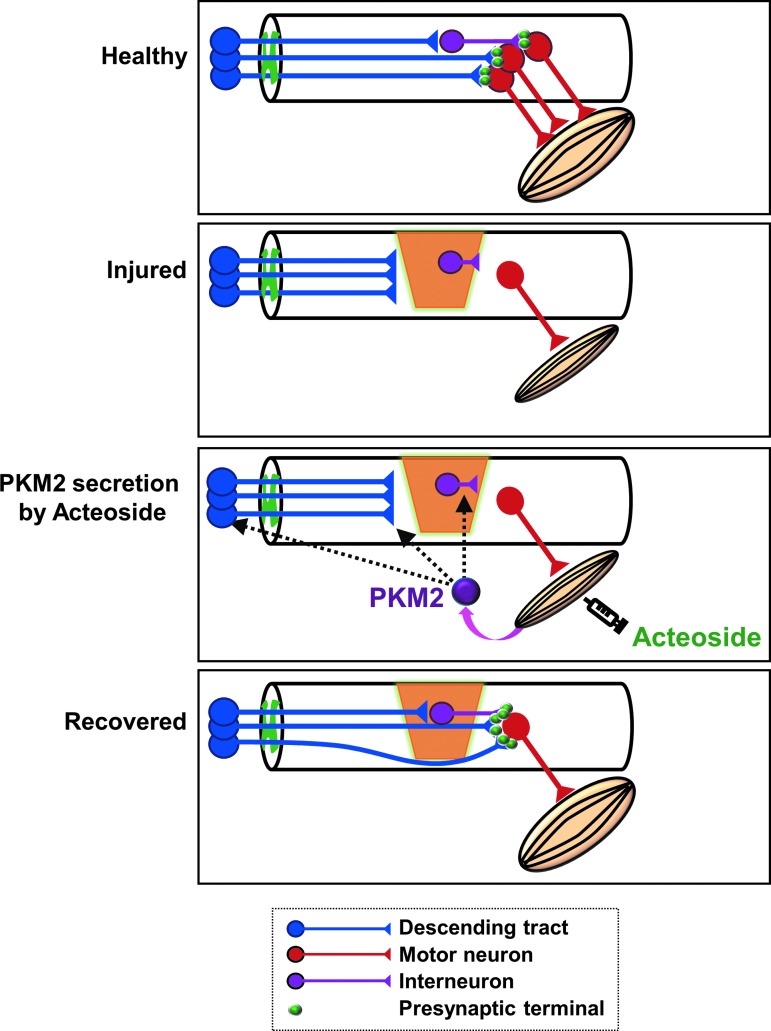
Schematic of the effects of acteoside and pyruvate kinase isoform M2 (PKM2) during the chronic phase of spinal cord injury. Color image is available online.

In conclusion, we found that acteoside improves skeletal muscle atrophy and locomotor dysfunction caused by chronic SCI, suggesting that it might be a promising therapeutic drug candidate for SCI. We also demonstrated that acteoside promotes the secretion of PKM2 from skeletal muscle cells, and that extracellular PKM2 induces axonal growth in cortical neurons and increases proliferation of skeletal muscle cells (Fig. 10). A strategy involving skeletal muscle-mediated therapy is a novel approach for chronic SCI, which might be effective in combination with other treatments.
